# Mothers’ Experiences of Care Coordination for Children with Disabilities: A Qualitative Study

**DOI:** 10.3390/children9060835

**Published:** 2022-06-04

**Authors:** Akemi Matsuzawa, Yuko Shiroki

**Affiliations:** 1 Department of Comprehensive Development Nursing, Graduate School of Health Sciences and Faculty of Medicine, Hokkaido University, Sapporo 060-0812, Hokkaido, Japan; 2 School of Nursing, Ibaraki Christian University, Hitachi 319-1295, Ibaraki, Japan; shiroki@icc.ac.jp

**Keywords:** children with disability, mother, primary caregiver, family, care coordination

## Abstract

Few studies have investigated the care coordination for children with disabilities and their families in Japan. Care coordination enhances the quality of care for these children and their families. This study explores mothers’ experiences of coordinated care provided to their children with disabilities and their families. We used a qualitative descriptive approach, conducting semi-structured interviews with 11 Japanese mothers/primary caregivers of children with disabilities to describe their experiences. Four main themes were identified: shared decision-making with key workers, receiving an assessment of the entire family, timely access to coordinated health care services, and a reduced psychological burden and empowerment of mothers. Our findings suggest that care coordination has multiple beneficial effects on children with disabilities and their families, including improving the outcomes. Further research should examine how high-quality care coordination can be provided for such children and their families.

## 1. Introduction

Care coordination is important for children with disabilities [1] and is a cornerstone of family-centered care [2,3]. In general, compared to typically developing children, children with disabilities face an increased risk of developing chronic physical, developmental, behavioral, or emotional conditions, and require additional health care and related services [4]. Therefore, as previous studies have reiterated, coordinated care and services are essential to ensure quality care for these children and their families [5].

In Japan, the need for coordinated care for children with disabilities is increasing as the number of children with disabilities is rising despite the declining birth rate. As of 2016, approximately 68,000 children suffer from physical disabilities and 214,000 children from intellectual disabilities [6]. The number of children receiving medical care is rapidly increasing too. The Ministry of Health, Labour and Welfare (MHLW) reported that 9403 were children dependent on medical technology in 2005 [7]. However, this number increased to 17,078 by 2015. Additionally, the number of ventilator-dependent children also increased from 264 in 2005 to over 3064 in 2015. Moreover, the health care system for children with disabilities in Japan is inadequate in both quality and quantity, which makes it difficult for all children with disabilities and their families to receive the high-quality services they need [8].

### 1.1. Background

The effect of care coordination for families of children with special health care needs (CSHCN) is well-documented and includes improvements in multiple outcomes, such as an increase in receiving family-centered care and experience of partnerships with professionals [9]. Additionally, care coordination for CSHCN meets almost all service-related needs [3] and fosters high family satisfaction [9,10,11], high primary- and specialty-care use, and low emergency room use [12]. Furthermore, parents of CSHCN receiving coordinated care exhibit better health and quality of life [13], and experience less impact on their employment [14]. In addition, the provision of coordinated care has been validated as one of the key quality indicators of the continuity of care for children with medical complexities [15].

Thus, pediatric care coordination is highly valued; however, it is often unavailable. In one study, 41% of parents reported that their children needed care coordination; however, of these, 31% did not receive effective coordination [16]. In another study, among children with medical complexity (CMC), 66.5% received no care coordination support, and 25.7% received clinic-based care coordination [17]; yet another study revealed that 68.2% of parents received care coordination, of whom 59.2% reported it to be adequate [9]. It is the quality of care coordination and not simply its availability to children and their families that contribute significantly to its effectiveness. Such results have been demonstrated among families raising CMC in Japan [8].

In Japan, social welfare services for children with disabilities were changed to a user-centered service system in 2003. In 2006, The Person with Disabilities Act encouraged and incentivized social welfare services for children with disabilities and their families. Until then, the system for access to social welfare services for their children was determined by the government. In 2012, the MHLW sought to strengthen support for children with disabilities who use social welfare services through care management services called “Soudan-Shien.” In this new system, these children receive the assessment, can avail care management activities, create a care plan, and ongoing monitoring when they apply for access to social welfare services. The care management services are typically conducted by professionals, such as social workers, social welfare workers, and registered nurses.

However, the new care management services system has not been adequately providing children with disabilities and their families with the services they need [18]. Additionally, the care management of social welfare services for these children is limited within the current system, which is more limited than the care coordination in developed countries. Moreover, till now, little research has been conducted on the care coordination experiences of these children and their families in Japan. Currently, studies of care coordination for children with disabilities and their families have found that care coordination by nurses is associated with the care burden of parents of children who are dependent on medical technology [19]. Moreover, providing high-quality care coordination is associated with the care burden of parents of children with disabilities [8].

The quantitative studies described above indicate that care coordination has been shown to be effective in reducing the care burden for these parents. However, the detailed experiences of care coordination from the perspective of these parents, especially mothers, remain unclear. To improve care coordination for children with disabilities and their families, the care coordination experiences of such children and families must be understood [20]. Therefore, it is necessary to qualitatively explore the subjective experiences and identify the positive outcomes of care coordination for these families.

### 1.2. Study Aim

We focused on the effective care coordination practices experienced by mothers raising children with disabilities at home. Consequently, this study aimed to explore such experiences of care coordination through mothers’ perceptions. Although several studies on care coordination outcomes used a quantitative approach, we explored care coordination experiences through a qualitative approach.

## 2. Materials and Methods

### 2.1. Participants and Recruitment

A qualitative descriptive analysis was conducted [21], using a purposive sampling strategy to recruit primary caregivers of children with disabilities. Participants were included in this study if: (1) they were the primary caregiver of a child with a disability, aged 1–18 years and living at home; and (2) their children received health care services from a health care service provider. Candidates were contacted through a center for care coordination for persons and children with disabilities and the visiting nurse station in Ibaraki Prefecture, Japan. Participants were recruited and interviewed between December 2013 and March 2014 by the primary author. Of the participants, one primary caregiver was biologically a grandmother. However, in this case, the biological mother was absent, so the grandmother played that role. Given that all other participants in the study were mothers, in this paper, we refer to all participants as “mothers.” The final sample comprised 11 mothers. We received an institutional review board exemption for this analysis through the Institutional Review Board of the University (Approval Number: 13–11). Written consent was obtained from all participants, including for audiotaping. Participants who signed the consent form were provided with a copy of the survey.

### 2.2. Data Collection

All the interviews were conducted by a primary author specializing in pediatric and family nursing and based on an interview guide. This included open-ended questions and semi-structured probes to particularly examine changes in daily life and physical and psychological well-being, in both the children and their families, since they began receiving home healthcare services. The interview guide was developed based on previous studies. It included questions assessing participants’ perceptions of their parenting in terms of the disabilities of their children, specifically regarding receiving care coordination.

Participants were interviewed in their homes, public centers, hospitals, or coffee shops, depending on their preferences. All interviews were conducted face-to-face with the primary author and lasted approximately 60–120 min. Participants were compensated with book coupons worth 2000 Japanese yen. The interview field notes provided additional contextual information. All interviews were audiotaped by the IC recorder and professionally transcribed verbatim for analysis.

### 2.3. Data Analysis

We used a qualitative descriptive approach. Data analysis was conducted on common concepts, and factors were coded and grouped into themes. The interview data underwent the following four major stages of coding and analysis.

First, the primary author repeatedly read all the transcripts to develop a detailed hierarchical numerical coding scheme. Subsequently, it was applied to all transcripts. After carefully reading the interview transcripts, we extracted several categories of care coordination experiences. Codes were examined, compared, merged, relabeled, and split as necessary. Second, we examined all extracted data and compared them to identify similar content areas. Furthermore, each step of the experience was labeled based on the data obtained from the interviews. Third, differences in opinions regarding coding were resolved through discussion. Following a team meeting with the research group, the coding structure was developed, and the data were reassembled. Finally, we discussed whether the names accurately reflected the experiences described in the data. Each researcher examined all the transcriptions, rechecking whether the identified skills fit the interview data. The second author read and coded a subset of the transcripts to ensure that the themes accurately represented the data.

## 3. Results

### 3.1. Participants’ Characteristics

We conducted 11 interviews with mothers of children with disabilities in Ibaraki Prefecture, Japan. The participants’ characteristics are presented in Table 1. These children were aged between 1–13 years and exhibited a variety of conditions. All these children were using some type of social service: five were using home nursing services, and five were using day services (i.e., social services in which children with disabilities can go to the center and receive care for their development).

### 3.2. Key Themes

The following themes emerged from the data analysis: (1) shared decision-making with key workers; (2) receiving an assessment of the entire family; (3) timely access to coordinated health care services; and (4) reduced psychological burden and empowerment of mothers. Each theme had between three and five sub-themes. The thematic findings with representational quotes are presented below (Table 2).

#### 3.2.1. Theme 1: Shared Decision-Making with Key Workers

Mothers mentioned the availability of key workers who helped them make decisions. They reported that the availability of a key worker who understood their child and family allowed smoother shared decision-making regarding child care and services. Some mothers expressed that the workers’ understanding of their children increased their confidence regarding child-rearing. The following are the testimonies of participants regarding shared decision-making with key workers.

“Before the care coordinator was available, I had to make care decisions despite not knowing how to care for my child. Now, I can perform shared decision-making with the care coordinator regarding child care and services.”

(ID1)

“Until now, I did not know which health care professional to ask regarding doubts about child care … and now I can ask the care coordinator immediately.”

(ID2)

“Although there are multiple service providers involved, it is helpful to have a single point of contact where they can coordinate services for my child.”

(ID8)

“I felt distressed about caring for my child alone …, but now the care coordinator helps me make child care choices through a shared care plan for my child. I think that parents should make final decisions on child care, but advice and information regarding child care must be provided.”

(ID10)

However, one mother, who does not have a care coordinator under the new care management system, explained that

“I have to make all the decisions myself… I’m very worried about what kind of life is best for my children and my family… I’m not sure if this is the right way.”

(ID6)

#### 3.2.2. Theme 2: Receiving an Assessment of the Entire Family

Mothers reported that the care coordinators also thought of other family members, such as mothers, siblings, and fathers. Furthermore, some mothers reported that coordinated care helped families in cases of emergency.

The care coordinator considered the lives of the whole family, especially problems experienced by the siblings of a child with a disability. One participant reported,

“I have to use services for a child with a disability in case of some school events for siblings. The care coordinator thought not only of the child with a disability but also of his/her siblings.”

(ID1)

Another participant mentioned,

“The care coordinator considered caring for both my child and me. I think that families need someone who will continue monitoring family caregivers’ health conditions, especially with regard to aspects of mental health.”

(ID2)

Yet another participant specified,

“When I fell sick in the past, I was unable to take care of my child … I contacted the care coordinator regarding these problems, and he/she immediately coordinated the additional health care services for my child.”

(ID7)

#### 3.2.3. Theme 3: Timely Access to Coordinated Health Care Services 

Mothers reported timely and coordinated access to health care services for their children. Care coordinators provided well-coordinated, integrated care involving several health care providers to continue the development of children with disabilities. Some mothers reported sharing their child’s health information through a care plan. Several mothers described their experiences with accessing and coordinating healthcare services:

“I am not very familiar with health care services in the community … if I did not have coordinated care services for my child … I would have to care for my child alone. I do not know what I would do without health care services for my child, but now the care coordinator supports me.”

(ID3)

“There are various health care services for children with disabilities in the community, but the local government does not provide this information to the families. The care coordinators provided me with information regarding respite care, the new health care service providers, and services my child might need in the future.”

(ID4)

“When I did not know what healthcare services were available for my child, care coordinators referred me to the facilities for children’s services that were accessible.”

(ID5)

“The care coordinator referred me to several healthcare service providers for my child. Also, I told the care coordinator that I wanted to get a wheelchair for my child.”

(ID8)

“I did not know what the needs of my child or family would be in the future. The care coordinator thought proactively, implementing well-coordinated care for my child and creating a stronger connection with the health care services.”

(ID11)

#### 3.2.4. Theme 4: Reduced Psychological Burden and Empowerment of Mothers

Mothers reported experiencing reduced psychological burden and greater empowerment upon receiving coordinated care. Some mothers felt that their care-related burden was relieved because the care coordinator supported their children. Others felt empowered by the support that they received. The mother’s narrative includes the following:

“I felt relieved because the care coordinator supported me. The care coordinator regularly visited my home and monitored my child’s condition.”

(ID2)

“I felt a reduced burden because the care coordinators and health care professionals provided thorough care and services for my children. Also, I felt empowered because the care coordinator planned goals for my children, and I strived toward achieving these goals with dedicated specialists.”

(ID10)

## 4. Discussion

This qualitative study explored Japanese mothers’ experiences of coordinated care for children with disabilities and their families. We found that mothers experienced shared decision-making and the availability of key workers, receiving a whole-family assessment, timely access to coordinated health care services, and reduced psychological burden and empowerment. These findings correspond with other studies, which reported that care coordination has multiple beneficial effects for children with disabilities and their families. To our knowledge, this is one of the very few studies that consider Japanese mothers’ care coordination experiences regarding their children with disabilities and their families.

In our study, most mothers reported access to timely and proactive services, which is consistent with the findings of previous studies. Access to health care and the timely use of personal-health services are essential for receiving services and optimal health outcomes. In particular, access to health care influences children’s physical and emotional development and their overall health and well-being [22]. Van Cleave et al. [23] examined care coordination in 12 pediatric practices, reporting that proactive care coordination activities were beneficial. To ensure easy access and optimize care, such services must be made available for all children with disabilities.

In Japan, services for children with disabilities are limited, especially for CMC. Previous studies have reported that parents consider healthcare services for their children as inadequate and experience difficulty in obtaining information concerning children’s services in-home care [18]. Although CMC requires a comprehensive and wide range of medical, psychosocial, educational, and support services from many agencies and programs, the current health system creates information-sharing barriers for families caring for CMC [24]. Thus, mothers face difficulty in gathering service information and arranging and integrating the needed health care services. Therefore, caregivers need help in effectively navigating health care services [20].

The results of the current study reflect that care coordinators play key roles for children with disabilities and their families. Traditionally, Japanese mothers have been the primary care coordinators; however, this role may unnecessarily burden mothers. Families caring for CMC experience difficulties, such as a limited understanding of their child’s medical complexity, the family’s psychosocial stresses, a dearth of parental motivation, and a lack of money or time [25]. As mentioned above, such children and families generally require health care services from a broad range of providers and systems, which means their care is often fragmented. Consequently, care coordinators can help such mothers and their families improve communication with care providers. The literature on care coordination for children has highlighted that establishing clear roles for families and providers is vital for enhancing relationships, improving communication, and ensuring continuity of care [26,27]. Further, in Japan, nurses’ provision of care coordination for families of technology-dependent children is associated with the physio-psychological burden of these families. High-quality care coordination is comprehensive and emphasizes cross-organizational relationships. Thus, care coordinators can play an important key role in supporting the entire family, reducing the burden on the primary caregiver, and ensuring continuity of care.

We also found that the mothers received decision-making support. The study participants reported that care coordinators discussed child-rearing goals with them, making shared decisions and care plans for children with disabilities. Shared decision-making is a key factor in family-centered care and is important for improving communication and partnership between families caring for CSHCN and health care providers [28,29]. The key aspect of care coordination is to develop a shared care plan between families, children, and the full range of medical and community care providers [30]. Therefore, such care plans can assure high-quality care coordination [31,32].

However, two mothers in this study neither availed of care management services nor had a specific care coordinator based on the current social welfare system. It is assumed that these mothers did not have care coordinators because the current system allows the use of the care management system only when social welfare services are used. In the results of this study, the children of both mothers were dependent on ventilators and were using home nursing services. In addition, one mother was worried about decision-making regarding her children and family. Therefore, professionals who care for those children and their families need to be involved with such children and their families in order to serve as key workers. Additionally, professionals should play a role in connecting children and their families to a care coordination system for them.

The findings in this study conform to those of other studies that have suggested that care coordination reduces mothers’ psychological burden and empowers them. In previous studies, high-quality care coordination for CSHCN was associated with parent satisfaction with care and services [9,11] and reduced caregiver burden [8,33]. Furthermore, parents of children with disabilities have reported negative psychological outcomes relative to parents of typically developing children. Raising children with disabilities also negatively impacts parents’ mental health [34], psychological well-being [35], and depressive symptoms [36]. Therefore, it is necessary for care coordinators to assess the health and life of not only children with disabilities but also their mothers, siblings, and fathers. However, only three mothers among the study participants had undergone an assessment of the entire family. Therefore, care coordinators should closely consider the health and well-being of mothers, siblings, and fathers. Further education to improve the assessment skills of care coordinators for these children and families is also needed.

## 5. Conclusions

This study explored the care coordination experiences of mothers of children with disabilities in Japan. The results indicate that care coordination has multiple positive effects on children and their families, notably the provision of timely and proactive coordinated care. There are intrinsic differences between the support required by adults and children; hence, children’s care coordination needs to change as they grow and develop [29]. Therefore, high-quality coordinated care is needed. Using a qualitative approach, we identified the positive aspects of coordinated care for children with disabilities; however, assessment of the whole family and shared decision-making is not always provided and the needs of children who do not receive care coordination services remain unfulfilled. Therefore, family-centered care coordination should be urgently provided for all such children and their families in Japan.

There are several limitations to this study. First, this study included only Japanese mothers from one prefecture in Japan. Second, participants’ reports may have been subject to recall bias and a tendency toward socially desirable answers. Finally, specific information on care coordination practices is limited. However, this study is among the few that reveal the experiences of the mothers of children with disabilities regarding care coordination, shortly after the introduction of the new care management system for children with disabilities and their families, in Japan.

The participants received coordinated care from social-welfare workers and registered nurses as care coordinators; the sample also included those who did not have a care coordinator for their children and families. In a previous study testing the effect of nurse-provided care coordination, advanced practice registered nurses played the role of care coordinators [37,38]. Thus, the quality of care coordination can vary widely. Further research should examine high-quality care coordination and evaluation outcomes for the whole family, including children with disabilities, fathers, and siblings.

## Figures and Tables

**Table 1:** Participants’ characteristics.

ID	Child’s Age (in Years)	Child’s Sex	Child’s Health Conditions	Primary Caregiver	No. of Family Members	Duration of Home Care	Care Coordinator’s Background (Professionals Who Play a Care Coordination Role)	Use of Social Service	Use of Care Management Service
1	9	Male	Developmental disorder	Mother	5	9 years	Social welfare worker	Day service, transfer service, home visit bathing service	+
2	10	Female	Neurological disorder and respirator-assisted	Mother	6	8 years	Social welfare worker	Doctor’s visit, home nursing services, home care service, transfer service, short stay service	+
3	4	Male	Developmental delay	Mother	3	4 years	Registered Nurse	Day service	+
4	10	Male	Neurological disorder	Mother	4	10 years	Social welfare worker	Day service	+
5	5	Male	Neurological disorder and respirator-assisted	Mother	8	3 years	Registered nurse	Home nursing services	―
6	6	Female	Neurological disorder and respirator-assisted	Mother	6	5 years	Registered nurse	Home nursing services	―
7	13	Female	Neurological disorder	Mother	4	10 years	Social welfare worker	Home nursing services, home care service, transfer service	+
8	2	Male	Neurological disorder and gastrostomy	Mother	3	3 months	Registered nurse	Home help service, transfer service	+
9	4	Female	Neurological disorder and respirator-assisted	Mother	4	1 month	Registered nurse	Home nursing services, home care service, home visiting rehabilitation	+
10	3	Male	Developmental disorder	Mother	3	3 years	Social welfare worker	Day service	+
11	13	Male	Developmental disorder	Grandmother (Separated from mother)	4	13 years	Social welfare worker	Day service	+

**Table 2:** Overview of themes and subthemes.

	Themes	Subthemes	ID
Theme 1	Shared decision-making with key workers	Obtain the health services by a key worker	ID1, 2, 3, 4, 6, 8, 9, 10, 11
		Think together	
		Support for decision-making as a parent	
		Monitoring of children and families	
Theme 2	Receiving an assessment of the entire family	Assessment of mothers	ID1, 2, 7
		Assessment of siblings and fathers	
		Assessment of an emergency of parent or sibling	
Theme 3	Timely access to coordinated healthcare services	Based on the growth and development of the child	ID1, 2, 3, 4, 5, 6, 7, 8, 9, 11
		Obtain information on healthcare services	
		Connecting children and their families to health services	
		Ensure coordination among healthcare service offices	
Theme 4	Reduced psychological burden and empowerment of mothers	Makes the mother feel better	ID1, 2, 3, 4, 10, 11
		Maternal emotional stability	
		Mother feels reassured	
		Desire to be a better parent	
		Better understanding of how to parent a child	

## Data Availability

The data sets used and analyzed during the current study are available from the corresponding author upon reasonable request.
